# Tracing the invisible mutant ADNP protein in Helsmoortel-Van der Aa syndrome patients

**DOI:** 10.1038/s41598-024-65608-x

**Published:** 2024-06-26

**Authors:** Claudio Peter D’Incal, Elisa Cappuyns, Kaoutar Choukri, Kevin De Man, Kristy Szrama, Anthony Konings, Lina Bastini, Kim Van Meel, Amber Buys, Michele Gabriele, Ludovico Rizzuti, Alessandro Vitriolo, Giuseppe Testa, Fabio Mohn, Marc Bühler, Nathalie Van der Aa, Anke Van Dijck, R. Frank Kooy, Wim Vanden Berghe

**Affiliations:** 1 Cognitive Genetics (COGNET) and Protein Chemistry, Proteomics and Epigenetic Signaling (PPES), Department of Biomedical Sciences, University of Antwerp, Antwerp, Belgium; 2 Massachusetts Institute of Technology, Cambridge, USA; 3 Neurogenomics, Human Technopole, Viale Rita Levi-Montacini 1, 20157 Milan, Italy; 4 High Definition Disease Modelling Lab, Stem Cell and Organoid Epigenetics, IEO, European Institute of Oncology, IRCCS, 20141 Milan, Italy; 5 Department of Oncology and Hemato-Oncology, University of Milan, Via Santa Sofia 9, 20122 Milan, Italy; 6 Friedrich Miescher Institute for Biomedical Research, Basel, Switzerland; 7 University of Basel, Basel, Switzerland; 8 Family Medicine and Population Health (FAMPOP), Department of Medicine and Health Sciences, University of Antwerp, Antwerp, Belgium; 9 Department of Experimental Oncology, European Institute of Oncology IRCCS, Via Adamello 16, 20139 Milan, Italy

**Keywords:** Activity-dependent neuroprotective protein (ADNP), Helsmoortel-Van der Aa syndrome (HVDAS), Antibody validation, Blocking peptide competition assay, *Adnp* knock-out cell line, Immunoprecipitation, Biotechnology, Developmental biology, Molecular biology

## Abstract

Heterozygous de novo mutations in the *Activity-Dependent Neuroprotective Homeobox* (*ADNP*) gene underlie Helsmoortel-Van der Aa syndrome (HVDAS). Most of these mutations are situated in the last exon and we previously demonstrated escape from nonsense-mediated decay by detecting mutant *ADNP* mRNA in patient blood. In this study, wild-type and ADNP mutants are investigated at the protein level and therefore optimal detection of the protein is required. Detection of ADNP by means of western blotting has been ambiguous with reported antibodies resulting in non-specific bands without unique ADNP signal. Validation of an N-terminal ADNP antibody (Aviva Systems) using a blocking peptide competition assay allowed to differentiate between specific and non-specific signals in different sample materials, resulting in a unique band signal around 150 kDa for ADNP, above its theoretical molecular weight of 124 kDa. Detection with different C-terminal antibodies confirmed the signals at an observed molecular weight of 150 kDa. Our antibody panel was subsequently tested by immunoblotting, comparing parental and homozygous CRISPR/Cas9 endonuclease-mediated *Adnp* knockout cell lines and showed disappearance of the 150 kDa signal, indicative for intact ADNP. By means of both a GFPSpark and Flag-tag N-terminally fused to a human ADNP expression vector, we detected wild-type ADNP together with mutant forms after introduction of patient mutations in *E. coli* expression systems by site-directed mutagenesis. Furthermore, we were also able to visualize endogenous ADNP with our C-terminal antibody panel in heterozygous cell lines carrying *ADNP* patient mutations, while the truncated ADNP mutants could only be detected with epitope-tag-specific antibodies, suggesting that addition of an epitope-tag possibly helps stabilizing the protein. However, western blotting of patient-derived hiPSCs, immortalized lymphoblastoid cell lines and post-mortem patient brain material failed to detect a native mutant ADNP protein. In addition, an N-terminal immunoprecipitation-competent ADNP antibody enriched truncating mutants in overexpression lysates, whereas implementation of the same method failed to enrich a possible native mutant protein in immortalized patient-derived lymphoblastoid cell lines. This study aims to shape awareness for critical assessment of mutant ADNP protein analysis in Helsmoortel-Van der Aa syndrome.

## Introduction

Activity-Dependent Neuroprotective Protein (ADNP) is a highly conserved protein essential for proper neurodevelopment and is involved in chromatin remodeling by constituting (1) the ATP-dependent BAF complex, (2) being part of the CDH4-ADNP-HP1 (ChAHP) complex, and interacting with (3) the CHD4 and BRG1^1–4^. The *ADNP* gene consists of six exons (five exons in previous nomenclature) of which only the last three translate into the functional protein^5^. ADNP contains several conserved protein domains such as an octapeptide sequence NAPVSIPQ (NAP) which accounts for the protein’s neuroprotective function^6^. Apart from NAP, ANDP contains additional functional domains. The bipartite nuclear localization signal (NLS) is necessary for transport towards the nucleus^7^. The homeobox domain, heterochromatin protein 1α (HP1α)-interacting PxVxL motif and ARKS motif indicate a DNA-binding potency and involvement in chromatin remodeling^1,8^. Recently, co-immunoprecipitation studies showed interaction of ADNP with autophagy regulator LC3^9^, histone deacetylase SIRT1^10^, and autism proteins EIF4E^11^ and SHANK3^12^, all of them indicating a molecular role of epigenetic mechanisms and autism. In 2014, de novo nonsense or frameshift mutations within the *ADNP* gene have been found to be cause Helsmoortel-Van der Aa syndrome (HVDAS; *OMIM 615873*), one of the most frequent forms of syndromic autism comorbid with intellectual disability and several extra-neurological deficiencies accounting for 0.2% of ASD cases worldwide. Helsmoortel-Van der Aa syndrome patients carry these mutations across the entire length of the gene, resulting in a premature stop in the transcript^13^. With almost all mutations situated in the last exon, we previously demonstrated escape from nonsense-mediated decay (NMD) with the presence of a mutant form of *ADNP* mRNA in the patients^14^. However, endogenous mutant proteins in *ADNP* deficient cell and animal models have not been visualized up until now.

Originally, ADNP was discovered as an essential gene for proper neuronal development with vasoactive intestinal peptide (VIP)-responsive potency^15^. Functions of VIP have been associated with electrical activity and protect neurons from apoptosis^16^. Upon stimulation, VIP is released by astroglia in the neurotrophic environment and screening for other potent neuropeptides has led to the discovery of activity-dependent neurotrophic factor (ADNF) which contains a 14-amino acid neuroprotective sequence (ADNF-14; VLGGGSALLRSIPA). Structure analysis of ADNF-14 with related peptides showed that its 9-amino acid core had a greater neuroprotective potency and was effective over a broader concentration interval. The novel ADNF14/9-like peptide carried the sequence NAPVSIPQ (NAP) and ADNF-9 screening in a mouse embryonic carcinoma (P19) cDNA library revealed it constitutes a small part of ADNP. Western blotting of a transformed bacterial extract showed a specific protein band at 90 kDa^6,17^. Furthermore, gene silencing experiments of *ADNP* in HT29 intestinal cancer cells resulted in a decreased expression of the protein, whereas p53 expression increased, thereby reducing cell viability. In the latter study, the observed ADNP protein band had a molecular weight of 114 kDa after incubation with an antibody raised against the C-terminal ADNP domain with peptide sequence ranging from aa989-1015^18^. A subcellular localization study in rat cortical astrocyte cultures showed ADNP-reactivity in nuclear fractions with minor reactivity in the cytoplasm. Here, protein expression of ADNP was obtained after incubation with a NAP-reactive antibody raised against the N-terminal portion aa261-354, resulting in a signal of 116 kDa. Antibody specificity was assessed by administration of recombinant ADNP in a 100 × excess to the primary antibody concentration. The band signal completely disappeared in the nuclear fraction, indicative of intact ADNP^19^. In 2007, ADNP was discovered as a component of the SWI/SNF chromatin remodeling complex after immunoprecipitation/mass-spectrometry based experiments. In this study, human recombinant ADNP was fused to an N-terminal GFP-tag, adding another 25 kDa to the molecular weight. In this way, recombinant ADNP could be separated from endogenous ADNP. In addition, two strategies were used for reliable ADNP detection such as (1) a GFP antibody for the recombinant ADNP-GFP fusion protein, (2) a C-terminal antibody recognizing both recombinant and endogenous ADNP. The GFP-ADNP plasmid construct was transfected into HEK293 cells and expression was analyzed by western blotting. GFP-ADNP was located at the expected molecular mass of 175 kDa, 25 kDa higher than the endogenous ADNP protein observed at 150 kDa. In a final experiment, a transient knock-down *ADNP* in HEK293 cells resulted in a reduction of the band signal at a molecular weight of 150 kDa^1^. Upon generation of a mouse model haploinsufficient for *Adnp*, western blotting was performed to assess the expression of ADNP in the cortex. A specific band signal was obtained after incubation with a C-terminal antibody (Bethyl Laboratories) raised against the sequence aa1050-1102. A significant reduction of ADNP was observed in *Adnp* haploinsufficient mice, although the molecular weight was not mentioned^20^. Later, a newer ADNP antibody from Chemicon was used in a western blot analysis of a HeLa protein lysate, where the antibody recognized multiple bands. Subsequently, HeLa cells were transfected with two different small interfering RNAs (siRNA), targeting *ADNP* expression. Western blot analysis resulted in disappearance of protein bands in the range of 95 kDa and higher, indicating that these signals are related to ADNP^21^. Another ADNP antibody commercialized by BD Biosciences was tested for its immunoreactivity in whole mouse brain protein lysates. The ADNP antibody showed a unique band signal at the predicted molecular weight of 124 kDa^22^. ADNP expression was also assessed in a transgenic mouse model for tauopathy, where western blot assessment at different stages in life (3–10.5 months of age) resulted in a specific age-dependent decline of ADNP in the cerebral cortex of Tau-Tg and control mice. Here, two antibodies raised against ADNP commercialized by BD Biosciences and Bethyl Laboratories both recognized a band signal at 150 kDa^23^. In 2020, the role of ADNP was investigated in mouse embryonic stem cells (mESCs) were mutations in the *Adnp* gene were introduced with CRISPR/Cas9 technology, targeting the 3’ end of exon 4. Western blot validation of wild type and *Adnp* mutant cell lines showed a decrease of ADNP expression in a knock-down cell line, whereas protein levels were totally absent in the endonuclease-mediated CRISPR line. The used ADNP antibody from R&D Systems showed a specific band signal at 150 kDa, which decreased after knock-down and was completely absent after CRISPR/Cas9^3^. In 2021, co-immunoprecipitation experiments on human neuroblastoma protein extracts revealed an indirect interaction of the NAD^+^-deacetylase enzyme SIRT1 with ADNP through the microtubule-end binding proteins EB1/EB3^10^. Here, ADNP immunoreactivity was observed in the eluted fraction with a molecular weight of 100 kDa using an N-terminal antibody (Santa Cruz) raised to aa1-138. Biological confirmation in a neuronal progenitor cell model however failed to show any ADNP immunoreactivity. Only recently, ADNP expression was investigated in Helsmoortel-Van der Aa patient-derived hiPSCs by a double antibody approach. Here, both an N- terminal and C-terminal antibody were used to detect ADNP in a control hiPSC cell line and a cell line mutant for the most frequent *ADNP* mutation pTyr719*. To determine the presence of a truncated protein, ADNP expression was assessed with western blotting in both isogenic control and mutant hiPSC lines. The Sarma Laboratory generated a C-terminal antibody raised against aa953-1102, which showed a decrease of ADNP in the mutant line. The specific ADNP band signal was observed at a molecular weight of 150 kDa, while the p.Tyr719* truncated fragment with a theoretical molecular weight of 80 kDa could not be detected with the N- terminal antibody^24^. In 2022, several SH3 domains were predicted in the amino acid sequence of ADNP, suggestive for cytoskeletal interactions^12^. To confirm these predictions, co-immunoprecipitation experiments were conducted using actin immobilized agarose beads in murine hippocampal protein lysates. Here, distinct band signals for SHANK3, a cytoskeleton-interacting protein with a theoretical molecular weight of 220 kDa, were observed around the 100 kDa marker. In addition, an extremely vague signal was observed in the eluted fraction after ADNP detection, resulting in immunoreactivity between the 135 and 180 kDa marker lanes amongst non-specific binding at lower molecular weights. Most recently, a murine neuroblastoma cell line was generated using CRISPR/Cas9 to link a GFP-tag sequence to the endogenous *Adnp* gene^25^. Moreover, two distinct heterozygous cell lines were created, displaying the p.Pro403* and p.Tyr718* *Adnp* mutations. Using a GFP antibody, western blotting on protein extracts derived from these cell lines generated a specific ADNP signal in the wild-type (175 kDa) condition together with lower molecular weights in the mutant lines, 71 kDa for p.Pro403* mutant and respectively 106 kDa for the p.Tyr718* mutant. Paralleling the exact experimental setting with an N-terminal ADNP antibody (Santa Cruz) aa1-138 resulted in multiple band signals with absence of a distinct ADNP signal.

It is clear from the above that visualization of ADNP protein by different antibody sources in western blotting is ambiguous, which has resulted in inconsistent results regarding ADNP detection with varying molecular weights from 60 to 175 kDa in independent studies of different labs (Table 1). In this study, we performed a critical cross-comparative study of existing and newly available ADNP antibodies in different model systems, with appropriate controls, to optimize the detection of ADNP for reliable and unequivocal evaluation of the protein stability and function. Following a proper characterization of the wild-type protein, the possible identification of a stable mutant ADNP protein could drastically impact the molecular mechanism of the syndrome, suggestive for either a loss-of-function or rather a gain-of-function disease mechanism.

**Table 1:** Overview of the reported ADNP antibodies with indication of the observed molecular weights and tested sample materials.

Antibody	Observed molecular weight	Sample material	Publication
ADNF-14 (Gozes Lab)	90 kDa with an additional band of 60 kDa	Mouse embryonic carcinoma (P19) cells	Bassan et al.^6^
ADNP aa989-1015 (Gozes Lab)	114 kDa	Human colorectal adenocarcinoma (HT29) cells	Zamostiano et al.^27^
CNAP aa261-354 (Gozes Lab)	116 kDa	Rat cortical astrocyte cultures	Furman et al.^19^
GFP antibody	175 kDa	Human embryonic kidney (HEK293T) cells transfected with expression construct	Mandel and Gozes^1^
ADNP aa1050-1102 (Bethyl Laboratories)	150 kDa		
ADNP aa1050-1102 (Bethyl Laboratories)	No indication of the molecular weight	Cortex of wild-type and heterozygous *Adnp* mice	Vulih-Shultzman et al.^20^
ADNP (Chemicon)	95 kDa–150 kDa	Human cervical epithelial (HeLa) cells	Gennet et al.^21^
ADNP (BD Biosciences)	124 kDa	Whole mouse brain	Nakamachi et al.^22^
ADNP (BD Biosciences)	150 kDa	Cerebral cortex of Tau-Tg mice	Schirer et al.^23^
ADNP aa953-1102 (Bethyl Laboratories)			
ADNP aa941-1102 (R&D Systems)	150 kDa	Mouse embryonic stem cells (mESCs)	Sun et al.^3^
ADNP aa104-300 (Bethyl Laboratories)	150 kDa	Human induced pluripotent stem cells (hiPSCs)	Yan et al.^24^
ADNP aa953-1102 (Sarma Laboratory)			
ADNP F9 (Santa Cruz)	Vague bands ranging from 35 to 180 kDa	Mouse hippocampus	Ivashko-Pachima et al.^12^
ADNP F9 (Santa Cruz)	100 kDa	Human neuroblastoma cell lines and neuronal progenitor cells	Hadar et al.^10^
ADNP F9 (Santa Cruz)	Wild-type ADNP: 175 kDa ADNP-Tyr718*: 106 kDa ADNP-Pro403*: 71 kDa	Genome edited heterozygous mouse neuroblastoma cell lines with endogenous ADNP fused to GFP	Ganaiem et al.^25^

ADNP has a predicted molecular weight of 124 kDa.

## Materials and methods

### Plasmid constructs

The pCMV3 expression vector encoding human wild-type ADNP fused to either an N-terminal GFPSpark or N-DYKDDDDK (Flag) tag was purchased from Sino Biological (HG11106-ANG; HG11106-NF). Mutations were inserted into these wild-type vectors by PCR mutagenesis using the Q5 Site-Directed mutagenesis kit (New England Biolabs; E0554S) according to manufacturer’s protocol. Mutagenesis primers were designed using the NEBaseChanger Tool (http://nebasechanger.neb.com/) and can be found in Table 2**.** DNA was purified from high-efficiency NEB 5-alpha Competent E. coli cells using the NucleoSpin Plasmid EasyPure Mini kit (Macherey Nagel; 740727.50) according to the manual. Mutations were verified using Sanger sequencing.

**Table 2:** List of modeled mutations and corresponding side-directed mutagenesis primers. The asterisk (*) indicates the incorporation of a premature termination codon following the *ADNP* mutation.

Mutation	cDNA base change	Forward primer	Reverse primer
Arg173*	C > T	GTGCACTTACtGAGATCCTCTTTATG	TTCTTACAGTAATAAACAGCTTG
Tyr719*	Duplication A	aCGAGCAAATGGAATTTCCC	TAAGTGCGCTTCACAGGT
Arg730*	C > T	ACTGAAAAAAtGAAAGTTAGATGATG	AAGGGAAATTCCATTTGC
Leu823Hisfs*6	Deletion TTGCTGGGTTTAA	CATGAAAGAATTAAATAAAGTCAAGCATG	CACGCCAGGCTTGTACTT
Asn832Lysfs*81	Deletion TAAA	GTCAAGCATGAGATGGATTTTG	TTTAATTCTTTCATGTTAAACCC

### Cell culture

HEK293T (ATCC; CRL-3216) and HeLa (ATCC; CCL-2) cell lines were purchased and cultured in DMEM (Gibco; 11965092), supplemented with 10% fetal bovine serum (Gibco; 26140079) and 1% penicillin/streptomycin (Gibco; 15070063). SHSY-5Y cells (ATCC; CCL-2) were purchased and cultured in DMEM/F12 (Gibco; 21331020), supplemented with 10% fetal bovine serum (Gibco; 26140079), 1% penicillin/streptomycin (Gibco; 15070063), 1% non-essential amino acids (Gibco; 11140050), 1% sodium pyruvate (Gibco; 11360070) and 1% sodium bicarbonate (Gibco; 25080094). Epstein-Barr virus transformed lymphoblastoid cell lines (LCLs) of healthy subjects and Helsmoortel-Van der Aa syndrome patients generated *in house* and were cultured in RPMI (Gibco; A1049101), supplemented with 15% fetal bovine serum (Gibco; 26140079), 1% penicillin/streptomycin (Gibco; 15070063), 1% sodium pyruvate (Gibco; 11360070), and 1% GlutaMAX (Gibco; 35050061). Wild-type mouse embryonic stem cells (mESCs) were derived from blastocysts (3.5 PC) of mixed 129-C57Bl/6 background and generated as described previously^2^. mESCs either complete *Adnp* deficient or carrying homozygous *Adnp* mutations, were *in house* generated by CRISPR/Cas9 and grown on gelatin-coated dishes in DMEM (Gibco; 21969-035), supplemented with 15% fetal bovine serum (Gibco; 26140079), 1 × non-essential amino acids (Gibco; 11140050), 1 mM sodium pyruvate (Gibco; 11360070), 2 mM l-glutamine (Gibco; 25030149), 0.1 mM 2-mercaptoethanol (Sigma-Aldrich; M6250), 1% penicillin/streptomycin (Gibco; 15070063) and homemade LIF. Wild-type HCT116 cells were purchased (ATCC; CCL-247) and heterozygous *ADNP* mutations were *in house* generated using CRISPR/Cas9. Cells were grown in DMEM (Gibco; 11965092), 10% fetal bovine serum (Gibco; 26140079, 2 mM l-glutamine (Gibco; 25030149), and 1% penicillin/streptomycin (Gibco; 15070063). Human induced pluripotent stem cell (hIPSCs) lines carrying two distinct *ADNP* mutations were *in house* generated by CRISPR/cas9 and cultured in DMEM/F12 (Gibco; 21331020) and mTeSR-1 medium (Stemcell Technologies; 100-0276) either on mitomycin C-inactivated mouse embryonic fibroblasts (MEFs) or on human-qualified Matrigel-coated plates after a limited number of passages. All cell lines were placed in a humidified 37%O2/5%CO2 incubator to reach optimal confluency. All human cell lines were obtained from consenting patients, guardians, tending clinicians, and parents and all methods were carried out in accordance with the guidelines and regulations of the University of Antwerp/University Hospital of Antwerp (UZA). All experimental protocol regarding these cell lines approved by the Ethics Committee of the Antwerp University Hospital.

### Transient transfection

HEK293T were transfected using 5 µg wild type or mutant N-terminal human ADNP GFPSpark or N-DYKDDDDK (Flag) tag plasmid constructs (Table 2) with Lipofectamine 3000 Transfection Reagent (Invitrogen; L3000008) in accordance with the manufacturer’s protocol. Transfection efficiency was about 70% in line with the manufacturer’s tested performance. Cells were harvested after 24 h of incubation for western blotting.

### Animals

Male C57BL/6JCr wild type mice were purchased from Charles River at the age of 10 weeks with a body weight of 25 g. Animals were socially housed with a maximum of eight animals in standard mouse cages (22.5 cm × 16.7 cm × 14 cm) at constant humidity and temperature in a 12/12 h light–dark cycle. Food and water were available ad libitum. No cage enrichment was applied. Ex vivo experiments were carried out with tissue samples at the age of 10 weeks. All conducted experiments were in compliance with the EU Directive 20,120/63/EU under ECD code 2015-83 after approval by the Animal Ethics Committee of the University of Antwerp. Male Wistar Han rats were purchased from Charles River and were a kind gift of Shivani Chotkoe (LEMP, Antwerp University). Rats were kept in a 12 h/12 h light–dark cycle with controlled temperature and humidity in enriched cages of two animals with free access to food and water. All conducted experiments were in compliance with the EU Directive 20120/63/EU under ECD code 2020-14 after approval by the Animal Ethics Committee of the University of Antwerp.

### Post-mortem tissues and subjects

Adult human brain (NB820-59177), human brain cerebellum (NB820-59180), human brain frontal lobe (NB820- 59186), and human brain hippocampus whole tissue lysates (NB820-59187) from a healthy 45-year-old male subject were purchased from Novus Biologicals. Cerebellar tissue of a deceased 9-year-old female was a kind gift from the Institute Born-Bunge vzw IBB NeuroBioBank of the University of Antwerp under HMTA20210040. Clinical information of a deceased six-year-old male carrying the heterozygous c.1676Adup/p.His559Glnfs*3 *ADNP* mutation was obtained under informed consent from the parents of the participant under B300201627322 and approved by the Ethics Committee of the Antwerp University Hospital. Cerebellar tissue was collected during autopsy and frozen in liquid nitrogen. Mutation validation was performed with Sanger sequencing using the forward primer 3′- TGATGTGCAAGTGCATCAGA-5′ and reverse primer 3′-TGTGCACTTCGAAAAAGAACAT-5′.

### Sample preparation

All cell lines were cultured to reach an optimal confluency prior to harvesting. Murine and rat brain tissues were dissected on ice, subsequently snap frozen in liquid nitrogen. Tissue was homogenized with the TissueRuptor II (Qiagen; 9002755) with mixing at lowest speed. Cerebellar tissue obtained from the post-mortem control child and our deceased Helsmoortel-Van der Aa syndrome patient was homogenized with the TissueRuptor II (Qiagen; 9002755) with mixing at lowest speed. Cells or tissues were lysed and homogenized in ice cold RIPA buffer (150 mM NaCl, 50 mM Tris, 0.5% sodium deoxycholate, 1% NP-40 and 2% sodium dodecyl sulfate), supplemented with the cOmplete, Mini, EDTA-free Protease Inhibitor Cocktail (Roche; 04693159001) together with PhosSTOP phosphatase inhibitor (Roche; 4906845001). Lysis occurred for one hour at 4 °C with agitation and cell debris was removed by centrifuging 30 min at maximal speed in a precooled centrifuge. The protein concentration was estimated with the Pierce BCA Protein Assay Kit (Thermo Scientific; 23225). Adult human whole brain, frontal lobe, hippocampus, and cerebellar protein lysates were purchased in a native lysis buffer, containing HEPES (pH 7.9), MgCl2, KCl, EDTA, Sucrose, Glycerol, Sodium deoxycholate, NP-40, and a cocktail of protease inhibitors.

### Western blotting

A total amount of 20 μg protein lysate was reduced with NuPAGE Sample Reducing Agent (Invitrogen; NP0009) in NuPAGE LDS Sample Buffer (Invitrogen; NP0007). Samples were heated for 10 min at 70 °C and subsequently loaded for separation using a Bolt 4 to 12%, Bis–Tris, 1.0 mm, Mini Protein Gels (Invitrogen; NW04120BOX) using Bolt MOPS SDS Running Buffer (Invitrogen; B0001) at 120 V. The Precision Plus Protein All Blue Prestained Protein Standard (Biorad; #1,610,373) was used for estimation of the molecular weight in all experiments. After separation, proteins were transblotted onto Amersham Protran Premium nitrocellulose membranes (Cytiva; GE10600008) using a Mini Trans-Blot cell (Biorad; 1703930) with a transfer buffer containing 25 mM Tris, 192 mM glycine and 20% methanol (pH 8.3). Successful protein transfer was checked with a Ponceau S solution (Sigma Aldrich; P7170). Nitrocellulose membranes were blocked with either 5% blocking-grade non-fat dry milk (Carl Roth; T145.4) or 5% BSA (Carl Roth; CP84.1) dissolved in TBST for one hour at room temperature with agitation. Antibodies used for ADNP detection are listed in Table 3. Primary antibodies were tested and optimized in 1:1000 to 1:5000 in either 5% blocking-grade non-fat dry milk/TBST or 5% BSA/TBST solution. Signal amplification was achieved by incubation with an appropriate HRP-conjugated immunoglobulins (Agilent) in a 1:2000 dilution in either 5% blocking-grade non-fat dry milk/TBST or 5% BSA/TBST solution. The signal was detected using the SuperSignal West Femto Maximum Sensitivity Substrate (Thermo Scientific; 34095) and visualized with the Amersham Imager 680 (Cytiva). GAPDH was used as a loading control for all the experiments. All raw western blot images and indicated protein weight markers are contained within the supplementary data of the manuscript.

**Table 3:** Tested ADNP antibody overview. The table indicates the used antibodies for this study together with the manufacturer and catalog number, host species, predicted reactivity, peptide sequence, and optimized dilutions for each western blot experiment.

Antibody	Product number	Host species	Reactivity	Immunogen sequence	Dilution
*ADNP (E-20) Polyclonal antibody*	Santa Cruz Biotechnology; sc-49344	Goat	Mouse, rat, and human	N-terminal antibody aa 1–138	1:1000
*ADNP (F-9) Monoclonal antibody*	Santa Cruz Biotechnology; sc-376674	Mouse	Mouse, rat, and human	N-terminal antibody aa 1–138	1:1000
*ADNP Polyclonal antibody*	Bethyl Laboratories; A300-104A	Rabbit	Human	C-terminal antibody aa 1050 and 1102	1:1000
*ADNP Polyclonal antibody*	St. John’s Laboratory; STJ91502	Rabbit	Human, mouse	N-terminal antibody aa 80–160	1:1000
*ADNP Polyclonal antibody*	Aviva Systems; ARP39186_P050	Rabbit	Mouse, rat, and human	N-terminal antibody aa 35–82	1:1000
*ADNP Polyclonal antibody*	Protein Technology; 17987-1-AP	Rabbit	Mouse, rat, and human	C-terminal antibody aa 757–1102	1:4000
*ADNP Polyclonal antibody*	Abcam; ab244286	Rabbit	Mouse, rat, and human	C-terminal antibody aa 850–1050	1:2500
*ADNP Polyclonal antibody*	Sarma Laboratory	Rabbit	Human, mouse	C-terminal antibody aa 953 -1102	1:1000
*GFP tag monoclonal antibody (GF28R)*	Invitrogen; MA5-15256	Mouse	Tag	GFP from the jellyfish *Aequorea Victoria* N-terminal peptide-KLH conjugated	1:1000
*DYKDDDDK Tag monoclonal antibody (FG4R)*	Invitrogen; MA1-91878	Mouse	Tag	Synthetic peptide (DYKDDDDK) coupled to KLH	1:1000
*HA Tag monoclonal antibody*	Sigma-Aldrich; H3663	Mouse	Tag	Clone HA-7 purified from hybridoma cell culture	1:1000
*ADNP (F-5) Monoclonal antibody*	Santa Cruz Biotechnology; sc-393377	Mouse	Mouse, rat, and human	N-terminal antibody aa 1–138	1:1000

#### Optimization of ADNP western detection strategies

During assessment of different antibodies, we optimized our western blot protocol. To ensure optimal transfer of ADNP from the SDS-PAGE gel to the nitrocellulose membrane, we tested three standard protocols that are programmed in the Power Blotter XL System (Invitrogen). These protocols differ in transfer time, with a *low molecular weight* protocol being the shortest and the *high molecular weight program* protocol lasting the longest. Successful transfer of three different programs was validated with a Ponceau S staining. The *low molecular weight* protocol showed a gradient of intense staining of the smaller proteins towards faint staining of bigger proteins. The opposite results were observed for the *high molecular weight* program. The *mixed-range molecular weight* protocol resulted in a uniform transfer of proteins. Since wild-type ADNP has a predicted molecular weight of 124 kDa, it can be considered as a high molecular weight protein. However, truncated forms of ADNP can be present of lower molecular weights^7^. Despite these optimizations using a semi-dry method, optimal transfer of *high molecular weight* proteins is rather achieved by a wet transfer method. Comparing both the signal intensity after a semi-dry transfer method and wet electroblotting, the ADNP signal was more intense after using a wet transfer tank (Biorad). We therefore continued our experiments with a wet transfer cassette (Biorad) using a transfer buffer, containing 25 mM Tris, 192 mM glycine with 20% methanol (pH 8.3). We tested the effect of different blocking buffers, antibody dilutions and washing buffers for all ADNP antibodies presenting with a non-specific signal. Comparing both TBST and PBST washing buffers resulted in a minimal effect on background staining, however non-specific bands were still present. Comparison of the two blocking solutions containing either 5% blocking-grade non-fat dry milk/TBST or 5% BSA/TBST solution showed best results and reduced non-specific binding after incubation with milk. For this reason, we continued using milk as blocking agent in all our experiments. The main obstacle in antibody testing remains presence of non-specific binding and background staining. Since the antibody concentration is also reported to play an important factor in the contribution of non-specific bands^26^, we next evaluated different dilutions for each ADNP antibody, as well as different dilutions of the appropriate secondary antibodies. Experiments were conducted with the antibody concentration providing the least number of non-specific bands without losing signal in the range of the predicted mass. Washing steps with TBST or PBST supplemented with Tween-20 in between antibody incubations were also strictly followed to further reduce non-specific binding and background staining. In a last optimization experiment, we fine-tuned the acquisition signal given by the ADNP antibody. Highly abundant proteins such as frequently used loading controls GAPDH and β-actin are clearly detectable with even the least sensitive chemiluminescent western blotting substrate at only seconds of exposure. However, ADNP was discovered as a low abundant protein at femtomolar concentrations which is experimentally harder to detect^6,27^. We visualize ADNP only after one to several minutes of exposure, depending on the cell or tissue type. When comparing the effect of horseradish peroxidase (HRP) substrate sensitivity for acquisition, we observed the most conclusive results after incubation with the more sensitive SuperSignal West Femto Maximum Sensitivity Substrate (Thermo Scientific). Despite multiple optimization strategies of our western blotting protocol, unambiguous ADNP-specific protein detection could not yet be improved with any of the antibodies of Santa Cruz, Bethyl Laboratories and St. John’s Laboratory.

#### Immunoprecipitation assay

Proteins were extracted from the HEK293T overexpression lysates (wild-type ADNP, p.Tyr719*, p.Arg730*, and p.Asn832Lysfs*81) and patient-derived LCLs (one control subject and three patients with the p.Ser404*, p. p.Leu831Ilefs*82, or p.Asn832Lysfs*81 *ADNP* mutation) using the Pierce Co-Immunoprecipitation Kit (Thermo Scientific; *26149*) according to the manufacturer’s protocol. Briefly, 10 μg of the IP-competent N-terminal ADNP F-5 antibody (Santa Cruz Biotechnology; *sc-393377*) was cross-linked to 50 μL of AminoLink Plus Coupling Resin. An amount of 500 μg of protein lysate was incubated overnight at 4 °C on an end-over-end shaker (VWR; *444*-*0503*). Protein elution was performed in 30 μL of 4 × Laemmli Sample Buffer (Biorad; *1610747*) supplemented with 10X NuPAGE Sample Reducing Agent (Invitrogen; *NP0004*). An amount of 20 μg of crude protein lysate was loaded as input sample, consequently the immunoprecipitated materials were also loaded in equal amounts of 20 μg. All protein samples were subjected to electrophoresis with equal loading volumes of 30 μL and were subsequently investigated by immunoblotting using the following primary antibodies (Table 3): anti-GFP (Invitrogen; *MA5-15256*) to detect recombinant ADNP in overexpression lysates, and the N-terminal ADNP antibody (Aviva Systems; *ARP39186_P050*) to visualize native ADNP in patient-derived LCLs. In addition, Pierce Control Agarose Resin (crosslinked 4% beaded agarose) was used as negative control (IgG). After western blotting (see above), proteins were visualized using the SuperSignal West Femto Maximum Sensitivity Substrate (ThermoScientific; *34094*) after labeling with the appropriate secondary antibody (Agilent) in a 1:2000 dilution.

### Statistical analysis

Data are presented as the mean ± S.E.M. from at least three independent experiments. Statistical analysis of the data was performed via a two-tailed student T-test assuming unequal variances in GraphPad Prism software, version 10; *p < 0.05, **p < 0.01, ***p < 0.001.

### Ethical approval

Experiments including C57BL6/JCr mice were in compliance with the EU Directive 20120/63/EU under ECD code 2015-83 after approval by the Animal Ethics Committee of the University of Antwerp. Experiments including Male Wistar Han rats were in compliance with the EU Directive 20120/63/EU under ECD code 2020-14 after approval by the Animal Ethics Committee of the University of Antwerp. Cerebellar tissue of a deceased 9-year-old child was a kind gift from the Institute Born-Bunge vzw IBB NeuroBioBank of the University of Antwerp under HMTA20210040 by the Ethics Committee of the Hospital Network Antwerp (ZNA) Middelheim and HogeBeuken, Antwerp, Belgium. Clinical information of a deceased six-year-old male carrying the heterozygous c.1676Adup/p.His559Glnfs*3 ADNP mutation was received under informed consent of the parents under B300201627322 and approved by the Ethics Committee of the Antwerp University Hospital, Antwerp, Belgium. HEK293T and HeLa cells were purchased from ATCC at low passage number with consistent use of cells below a passage number of 15. Wild-type mouse embryonic stem cells were derived from blastocysts (3.5 PC) of mixed 129-C57Bl/6 background and cultivated on feeder cells (37 °C, 7% CO2), subsequently CRISPR/Cas9-engeneered to generate heterozygous and complete *Adnp* knockout lines^2^. HCT116 cells were purchased from ATCC and CRISPR/Cas9-engeneered with extensive validation in the laboratory of Marc Bühler. Human iPSCs were generated from skin fibroblasts donated by consenting patients and guardians/physicians in the laboratory of Giuseppe Testa/Frank Kooy. Lymphoblastoid cell lines were generated from peripheral blood lymphocytes donated by consenting patients and guardians/physicians in the laboratory of Frank Kooy.

## Results

### ADNP western analysis with common commercially available antibodies results in non-specific bands with doubtful ADNP-specific signal

**Figure 1:**
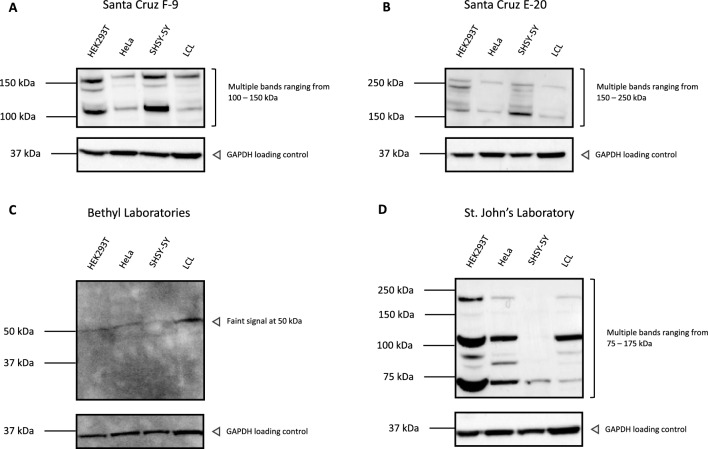
Common commercially available ADNP antibodies give rise to non-specific binding. HEK293T, HeLa, SHSY-5Y and a lymphoblastoid control cell line (LCL) were lysed in RIPA buffer and used as protein samples for the assessment of the published ADNP antibodies. Samples were blocked and incubated in 5% blocking-grade non-fat dry milk/TBST with the optimized dilution listed in Table 3. The predicted molecular weight of ADNP is 124 kDa. However, only non-specific signals were detectable. GAPDH was used as a loading control. The datasheet of the tested antibodies indicated that whole or nuclear extracts from HeLa cells should be used as a positive control, which fails to raise a reliable ADNP signal in all tested antibody conditions.

Different primary ADNP antibodies mentioned in literature were tested for their ability to detect wild-type ADNP by means of immunoblot analysis. We started a preliminary evaluation with the following antibodies: goat polyclonal ADNP (E-20) antibody (Santa Cruz Biotechnology), mouse monoclonal ADNP (F-9) antibody (Santa Cruz Biotechnology), and rabbit polyclonal ADNP antibody (Bethyl Laboratories). Wild-type human ADNP has a predicted molecular weight of 124 kDa based on its amino acid sequence^14^. However, a clear signal at the predicted molecular weight could not be detected as multiple bands were visible ranging from 50 to 250 kDa in total protein lysates from HEK293T, HeLa, SHSY-5Y and a control lymphoblastoid cell line (LCL) (Fig. 1A–C). Besides, we also selected a more recently developed ADNP antibody from the St. John’s Laboratory (STJ91502) to assess its western blot’s potency. Previous publications of Furman et al.^19^ and Gennet et al.^21^ reported that antibodies for ADNP detection present multiple bands after western blotting^19,21^. This observation also applied for the St. John’s Laboratory ADNP antibody (STJ91502) tested here. The antibody only recognizes multiple bands ranging from 75 to 175 kDa in total protein lysates from HEK293T, HeLa, SHSY-5Y and LCL, only when using the SuperSignal West Femto Maximum Sensitivity Substrate (Fig. 1D). No visible bands were observed after incubation with a normal range Pierce ECL Western Blotting Substrate. Although the specific ADNP signal might be present alongside non-specific signals, use of the St. John’s Laboratory antibody failed to unambiguously detect ADNP expression levels. In summary, we were not able to unambiguously detect ADNP with the antibodies from Santa Cruz, Bethyl Laboratories and St. John’s Laboratory. The signals obtained after incubation with these antibodies resulted in a non-specific signal and none of them correspond to protein’s predicted molecular weight of 124 kDa.

### Improved in vitro ADNP-specific protein detection by a new polyclonal N-terminal ADNP antibody (Aviva Systems)

**Figure 2:**
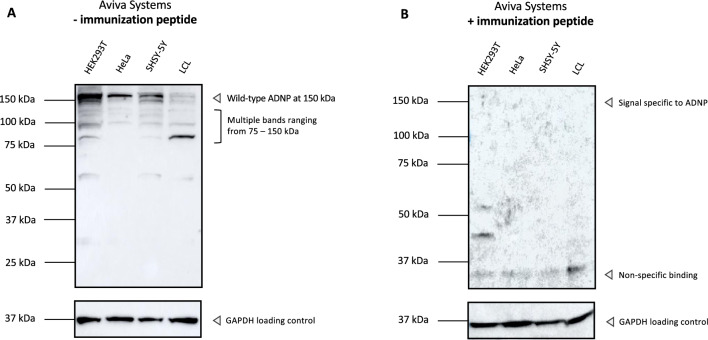
Verification of the specificity of an N-terminal ADNP antibody (Aviva Systems) by performing a blocking peptide competition assay. (**A**) HEK293T, HeLa, SHSY-5Y and a control lymphoblastoid cell line (LCL) were lysed in RIPA buffer and used as protein samples for the assessment of N-terminal antibody of Aviva systems in a 1:1000 dilution. GAPDH was used as a loading control. The predicted molecular weight of ADNP is 124 kDa. The antibody recognizes ADNP specifically at 150 kDa in HEK293T, HeLa and SHSY-5Y cell lines, but a faint signal ranging from 75 to 150 kDa in the control LCL. **(B)** Western blot analysis of the blocking peptide competition assay. Supplementation of the immunization peptide in a 5 × excess to antibody concentration reduced the signal detected at 75- 150 kDa in all tested cell lines. Non-specific binding was detected after use of the immunization peptide presenting as a faint signal below the 37 kDa marker.

Over the years, commercialization of antibodies has been incorporated in current biomedical research, with antibody production on a large scale even upon request^28^. Recently, Aviva Systems developed an N-terminal antibody raised against the amino acids 35–82 of a human recombinant ADNP peptide (ARP39186_P050) with a predicted reactivity in human, mouse, and rat samples. Incubation of this new N-terminal ADNP antibody with total protein lysates from HEK293T, HeLa, and SHSY-5Y resulted in a specific signal at 150 kDa. Additionally, ADNP was also detected with a faint signal at 150 kDa in the control LCL with an additional range of 75–150 kDa signals. Besides the specific 150 kDa signal, multiple signals were observed below the 150 kDa marker, suggesting either instability of the protein resulting in a degrading smear (Fig. 2A). In the past, researchers have tackled ADNP antibody specificity using blocking peptides or siRNA to interfere with ADNP expression^19,21^. We employed a similar strategy to assess the specificity of the N-terminal antibody of Aviva Systems. The rationale is that adding the immunization peptide to the antibody solution will decrease the specific antibody binding on the nitrocellulose membrane, resulting in a reduced signal. To assess the specificity of this antibody, we purchased the antigen for host immunization, which was commercially available in order to perform a blocking peptide competition assay. By comparing experimental conditions using only the ADNP antibody solution versus supplementation of the corresponding immunization peptide in a 5 × excess to the antibody concentration, we could verify disappearance of the western signal after incubation with the immunizing peptide as the signal specific for ADNP in total protein lysates from HEK293T, HeLa, SHSY-5Y and the control LCL derived from a control subject (Fig. 2B). We suspect that the antibody recognizes the ADNP epitope in the range of the molecular weight of protein, which was reduced after the blocking peptide competition assay. However, the presence of multiple band signals, especially in the control LCL, without a clear ADNP signal highlights that detection of the protein is ambiguous in some in vitro sample lysates.

### Improved in vivo ADNP-specific protein detection by a new polyclonal N-terminal ADNP antibody (Aviva Systems)

**Figure 3:**
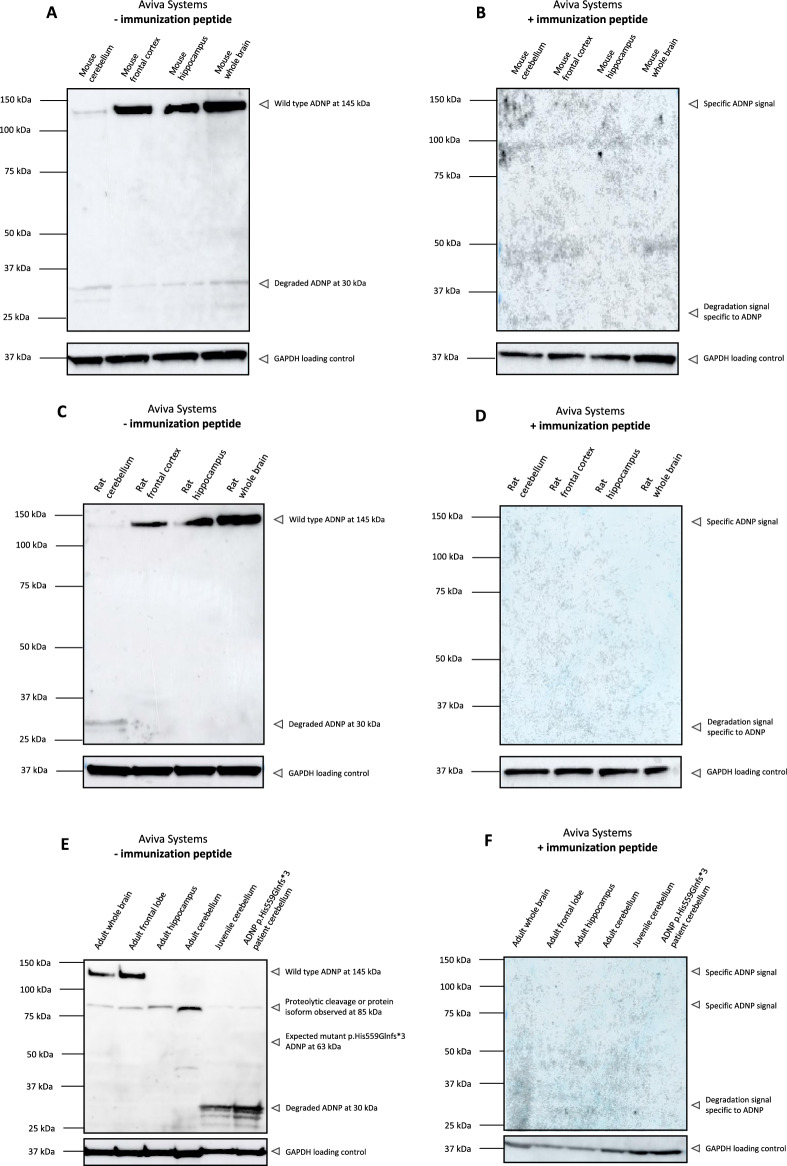
A polyclonal N-terminal ADNP antibody from Aviva Systems detects ADNP specifically in murine and rat tissues and suggests proteolytic processing of the protein in the human brain. Cerebellum, frontal cortex or lobe, hippocampus and whole brains of control mice, rats and humans were lysed in RIPA buffer and used as protein samples for the assessment of N-terminal antibody of Aviva systems. **(A**–**C)** The predicted molecular weight of ADNP is 124 kDa. The antibody recognizes ADNP in a range of 145 kDa with** (E)** additional lower mass signal of 85 kDa in all human brain regions.** (B**–**D**–**F)** Western blot analysis of the blocking peptide competition assay. Supplementation of the immunization peptide in a 5 × excess to antibody concentration reduced the signal observed at 145 kDa in all tested cell lines. Importantly, the 85 kDa band suggestive for proteolytic cleavage as well as degraded ADNP signal disappeared completely after immunization peptide supplementation. GAPDH was used as a loading control.

Since we observed a decrease in the supposed ADNP signal after blocking with the immunization peptide in cell lines with the N-terminal antibody of Aviva Systems, we challenged the antibody’s capacity to detect a specific ADNP signal in different brain tissue regions of mouse, rat, and human origin. In line with the previous experiments, we performed the blocking peptide competition assay as assessment for the antibody specificity (Fig. 3). In the different mouse and rat brain regions a very clear ADNP signal can be observed at a molecular weight of 145 kDa, which totally disappeared after supplementation with the blocking peptide (Fig. 3A–D). Expression was the lowest in the cerebellum compared to frontal cortex, hippocampus, and whole brain. These findings are indicative for reliable ADNP detection with the N-terminal antibody of Aviva Systems in different rodent brain regions. However, in post-mortem human brain lysates the situation seems less straightforward. Here, a clear ADNP signal appears in whole brain and frontal lobe lysates with a molecular weight of 145 kDa. Surprisingly, an additional signal of 85 kDa was observed in human brain only, which disappeared in the blocking peptide condition. This suggests that the 85 kDa band signal contains an epitope recognized by the N-terminal antibody, indicative for a proteolytic cleavage product of ADNP or an unknown protein isoform. Comparison of a post-mortem age-matched cerebellum of a healthy subject to a seven-year-old deceased boy carrying the heterozygous *ADNP* mutation p.His559Glnfs*3 showed no evidence for wild-type ADNP detection, expected at 150 kDa, possibly attributed to the long post-mortem interval. Besides, a clear degradation signal was present at the molecular weight of 30 kDa. In addition, we were unable to detect a mutant ADNP protein with a predicted molecular mass of 63 kDa **(**Fig. 3E,F).

### Different C-terminal ADNP antibodies show discrete specific immunoblot signals in a broad range of 35–145 kDa, significantly overlapping with the immunoblot pattern of the N-terminal antibody (Aviva Systems), suggestive of ADNP degradation mechanisms

**Figure 4:**
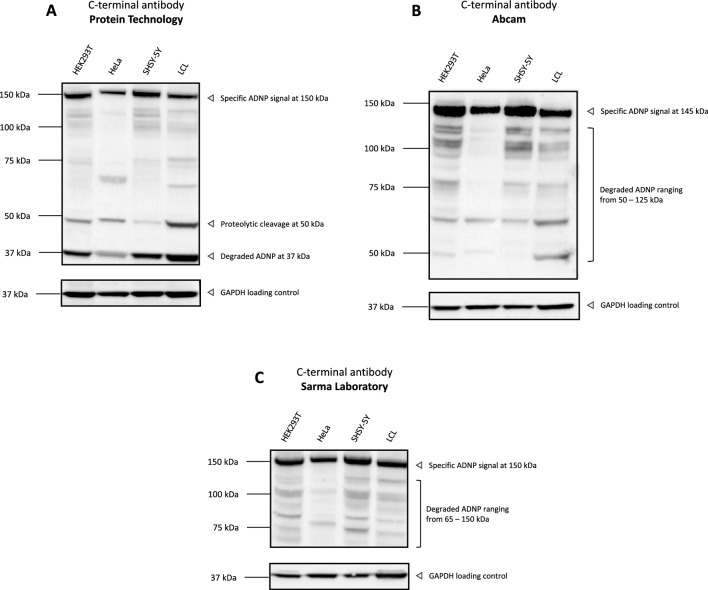
Three independent commercially available C-terminal polyclonal ADNP antibodies detect ADNP specifically in different in vitro sample materials and show clear instability of the protein. HEK293T, HeLa, SHSY-5Y and a lymphoblastoid cell line (LCL) were lysed in RIPA buffer and used as protein samples for three different C-terminal ADNP antibodies. GAPDH was used as a loading control. The predicted molecular weight of ADNP is 124 kDa. All the tested antibodies recognized ADNP with a molecular weight of 150 kDa. Samples were blocked and incubated in 5% blocking-grade non-fat dry milk/TBST with the optimized dilution listed in Table 3.

ADNP was initially discovered in the neurotrophic environment of glial cells, and hence can therefore potentially be secreted in the extracellular matrix^6^. Many secreted proteins often undergo proteolytic processing by proteases, which can cause inconclusive western blot results^29^. For this reason, we additionally tested several C-terminal antibodies raised against different amino acid residues of the ADNP protein, because N-terminal antibody detection can often be difficult because of epitope retention in the hydrophobic core of the protein which is not always fully exposed^6,30^. The polyclonal C-terminal antibody of Protein Technology was raised against amino acids 757–1102 of ADNP and shows reactivity with mouse, rat, and humans. The antibody recognized a specific ADNP signal at 150 kDa in total protein lysates from HEK293T, HeLa, SHSY-5Y and a control LCL. However, additional signals were observed at 50 kDa and 37 kDa, possibly due to proteolytic cleavage, instability of the protein, or non-specific binding (Fig. 4A). Next, a polyclonal C-terminal antibody of Abcam was raised against amino acids 850—1050 of ADNP which shows immunoreactivity with mouse, rat, and humans. The antibody recognized a specific ADNP signal at 150 kDa in total protein lysates from HEK293T, HeLa, SHSY-5Y and a control LCL, contributing to the most intense signal of the three different C-terminal antibodies. However, clear signals were observed ranging from 50 to 125 kDa, suggestive of instability, degradation, or non-specific binding of the protein (Fig. 4B). Lastly, we implemented a polyclonal C-terminal antibody of the Sarma Laboratory, raised against amino acids 953—1102 with murine and human reactivity^24^. The antibody also showed a clear ADNP signal at 150 kDa in total protein lysates from HEK293T, HeLa, SHSY-5Y and a control LCL. A smear of bands ranging from 65 to 150 kDa was detected and suggests decay of the protein (Fig. 4C). These results indicate that C-terminal antibodies raise reliable western blot results and uniformly conclude a molecular weight of 150 kDa of the non-processed ADNP protein in different in vitro sample materials.

**Figure 5:**
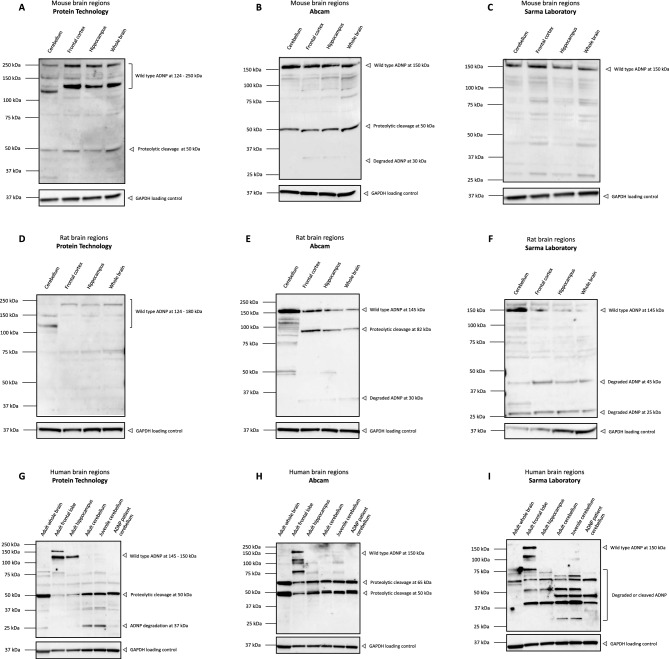
Different C-terminal ADNP antibodies detect ADNP in the range of 150 kDa and suggest proteolytic processing of the protein in the brain. Cerebellum, frontal cortex or lobe, hippocampus and whole brains of control mice, rats, and humans were lysed in RIPA buffer and used as protein samples for the assessment with three C-terminal antibodies with the optimized dilutions listed in Table 3. GAPDH was used as a loading control. The predicted molecular weight of ADNP is 124 kDa. (**A**)**C**) Murine samples indicate detection of ADNP in the range of 150 kDa with bands suggesting proteolytic processing at 50 kDa. (**D**–**F**) Rat samples indicate detection of ADNP in the range of 150 kDa with bands indicating proteolytic processing at 82 kDa after incubation with the C-terminal Abcam antibody. (**G**–**I**) Human brain samples indicate detection of ADNP at different molecular weights of 124 – 150 kDa in the adult frontal lobe and hippocampus and highlight the antibody differences in detection of ADNP. The three tested antibodies showed strong band signals at lower molecular weights, which could indicate proteolytic cleavage or degradation of the protein.

Since ADNP protein could clearly be detected in different cell line lysates, antibodies were next evaluated for a reliable ADNP detection in in vivo sample materials. For this reason, we challenged the C-terminal antibody capacities to different brain regions of mouse, rat, and human origin, in parallel with the previously tested N-terminal antibody of Aviva Systems. Unfortunately, none of the tested commercial C-terminal antibodies provides peptide used for immunization and therefore no blocking strategy could be performed for verification. First, we performed a western blot analysis with these distinct C-terminal ADNP antibodies on different murine brain regions. Here, the C-terminal ADNP antibody of Protein Technology detected multiple bands ranging from 124 to 250 kDa together with a possible proteolytic cleavage band of 50 kDa (Fig. 5A**)**. The C-terminal antibody of Abcam showed a clear ADNP signal of 150 kDa with a suggested proteolytic cleavage band of 50 kDa (Fig. 5B**)**. The C-terminal ADNP antibody of the Sarma Laboratory also indicated a clear band signal of 150 kDa (Fig. 5C**)**. Next, we performed a western blot analysis with the same C-terminal antibodies on different rat brain regions. The C-terminal ADNP antibody of Protein Technology detected multiple faint bands ranging from 124–180 kDa in the tested brain regions (Fig. 5D**)**. The Abcam antibody showed a specific ADNP signal at 150 kDa together with a possible proteolytic cleavage band of 82 kDa (Fig. 5E**)**. The Sarma Laboratory antibody which is predicted to only react with mouse and human species, also showed a specific ADNP band in the rat cerebellum at 150 kDa with a fading signal in the other brain regions. In addition, visibility of multiple degraded band signals also suggests significant involvement of protein decay mechanisms (Fig. 5F**)**. Ultimately, we subjected our selection of C-terminal antibodies to different adult and juvenile brain regions. The antibody of protein technology only detected wild type ADNP at 124—150 kDa in the adult frontal lobe and hippocampus, but not in the other adult or juvenile brain regions. Additionally, a clear proteolytic cleavage band signal of 50 kDa was observed in all tested brain lysates (Fig. 5G**)**. Contrasting to the protein technology antibody, the Abcam antibody showed a clear ADNP signal at 150 kDa in the adult frontal lobe, but not in the other regions. However, two very strong band signals were observed in all the tested conditions at 65 kDa, respectively at 50 kDa, indicative for proteolytic processing of the protein (Fig. 5H). Like the observed ADNP signal at 150 kDa after incubation with the Abcam antibody, the Sarma Laboratory antibody was also able to capture a specific signal at the same molecular weight in the adult frontal lobe. A strong ADNP degradation signal can be visualized at the molecular weight of 37–75 kDa (Fig. 5I). These results highlight the complexity of ADNP detection and indicate that these results are antibody dependent. However, a combination of the N-terminal antibody together with C-terminal antibodies confirms a reliable molecular weight of ADNP at 145–150 kDa. We strongly recommend the implementation of these N-terminal and C-terminal antibodies in future research to monitor ADNP protein detection.

### Verification of ADNP-specific immunoblot detection strategies in a CRISPR/Cas9 Adnp knockout mESC cell model

**Figure 6:**
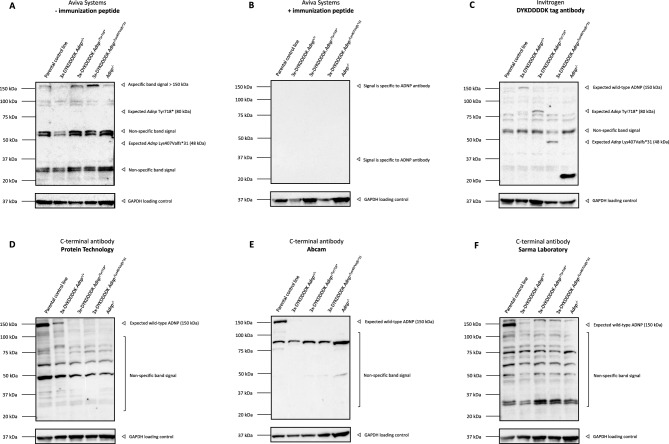
Unambiguous detection of ADNP using homozygous CRISPR/Cas9 endonuclease-mediated *Adnp* knockout cell lines. mESCs containing either wild-type, homozygous mutants, or complete *Adnp* knockout were lysed in RIPA buffer and used as protein samples for the assessment with an N-terminal ADNP, 3x-DYKDDDDK, and C-terminal ADNP antibodies with the optimized dilutions listed in Table 1. GAPDH was used as a loading control. The predicted molecular weight of ADNP is 124 kDa. (**A**) The N-terminal antibody (Aviva Systems) recognizes ADNP in a range above its observed 150 kDa molecular weight with additional lower mass signal of 37—65 kDa in *Adnp* homozygous and parental control mESCs. (**B**) Supplementation of the immunization peptide in a 5 × excess to antibody concentration reduced all signals observed mESC lines, indicating that the N-terminal antibody does not bind ADNP specifically in mESCs. (**C**) Detection of wild-type and homozygous *Adnp* mutants by means of a C-terminal 3x-DYKDDDDK (Flag) epitope tag. Wild-type ADNP was detected in at 150 kDa in the C-terminal 3x-DYKDDDDK CRISPR/Cas9 engineered mESC line using a DYKDDDDK antibody. Truncated ADNP mutants, p.Tyr718* and p.Lys407Valfs*31, were detected at a lower molecular weight of 80 kDa, respectively 48 kDa. (**D**–**F**) Wild-type ADNP detection by means of three different C-terminal antibodies in mESC lines. Wild-type ADNP was detected with a strong signal at 150 kDa in the parental control line with a rather decreased signal in the C-terminal 3x-DYKDDDDK CRISPR/Cas9 engineered mESC line. Disappearance of the 150 kDa band was observed in the mESC line with complete Adnp homozygosity, indicating a reliable molecular weight of 150 kDa for ADNP.

In mice, *Adnp* knockout homozygosity has detrimental effects on neural tube closure, leading to early embryonic death at day E8.5-E9^31^. However, complete knockout of *Adnp* in mouse embryonic stem cells (mESCs) affects morphology, but not viability with cells expressing pluripotency markers. To study the molecular function of ADNP, deficient mESCs were created with CRISPR/Cas9 technology by (1) insertion a 3 kDa C-terminal Flag-AviTag (3x-DYKDDDDK) at the endogenous *Adnp* gene, (2) introduction of the homozygous mutations, p.Tyr718* and p.Lys407Valfs*31, fused to a C-terminal Flag-AviTag (3x-DYKDDDDK), and (3) complete *Adnp* knockout homozygosity, by targeting eight base pairs downstream of the ATG start codon in exon 3 and right downstream of the stop codon in exon 5 to cut out the entire coding region^2^. Immunoblotting of total protein lysates of a parental control cell line and *Adnp* homozygous cell lines with the N-terminal antibody (Aviva Systems) resulted in detection of a band above the molecular weight marker of 150 kDa. Moreover, none of the two ADNP mutant proteins p.Tyr718* and p.Lys407Valfs*31 were detected at a lower expected molecular weight of 80 kDa and 48 kDa respectively. In contrast, strong bands were observed at lower molecular weights of 65 kDa and 37 kDa, which are also observed in the complete homozygous *Adnp* knockout condition, indicating non-specific binding (Fig. 6A). Administration of the blocking peptide caused complete loss of all observed signals after incubation with the N-terminal Aviva Systems antibody and may indicate remaining cross-reactive epitopes in homozygous *Adnp* knockout mESC cells, for example of ADNP2^32^, and non-specific binding (Fig. 6B). To alternatively demonstrate stable translation of 3x-DYKDDDDK (Flag) wild-type ADNP and mutants, we performed the same western blot with incubation of a DYKDDDDK (Flag)-tag antibody. Here, we could confirm presence of wild-type ADNP protein around 150 kDa amongst a strong background signal together with the introduced mutants at the predicted lower molecular weights (Fig. 6C). To assess the molecular weight of ADNP, we tested three different C-terminal antibodies on the parental and CRISPR/Cas9 engineered mESC lines. Although none of the C-terminal antibodies contains the epitope for mutant ADNP detection, we questioned whether the wild-type band signal would disappear after antibody incubation in homozygous mutant cells. In fact, all C-terminal ADNP antibodies were able to detect wild-type ADNP with a strong signal of 150 kDa in the parental lines, respectively a faint signal in the CRISPR/Cas9-mediated cell line with wild-type ADNP 3x-DYKDDDDK. Although ADNP mutants could not be visualized with our C-terminal antibody panel, we observed complete disappearance of the wild-type ADNP signal in the *Adnp* homozygous knock-out condition, again confirming an observed molecular weight for endogenous ADNP of 150 kDa (Fig. 6D-F). In this section, we created *Adnp* mESCs containing either wild-type, homozygous mutants, or complete *Adnp* knockout to confirm its molecular weight and to verify the specificity of different N-terminal and C-terminal antibodies (described above). A combination of both antibodies raised against different ADNP epitopes as well as a complete disappearance of the ADNP signal in an *Adnp* homozygous knockout cell line provide definitive proof for an observed molecular weight of 150 kDa for wild-type ADNP. The presence of band signals at lower molecular weights in the knock-out conditions clearly indicates non-specific protein band as detected with the N-terminal ADNP antibody in mESCs.

### Unambiguous detection of ADNP with an N-terminal GFPSpark and N-DYKDDDDK (Flag) tag expression vector: a dual antibody approach for reliable ADNP detection

**Figure 7:**
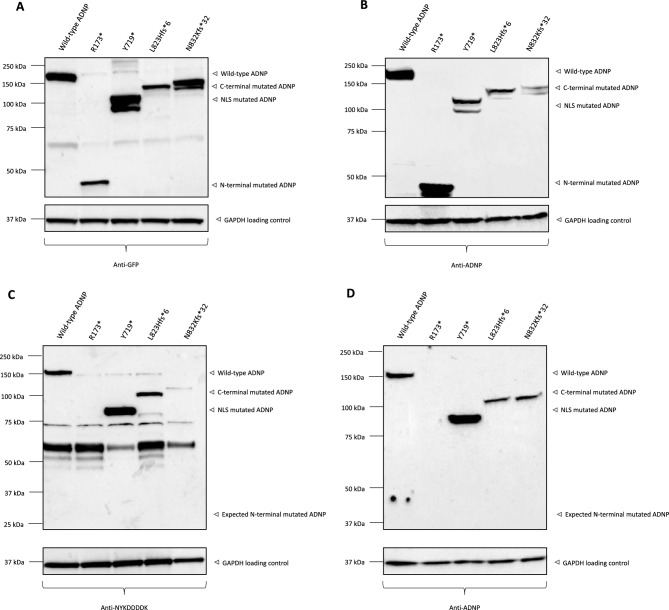
Unambiguous detection of ADNP using an N-terminal GFPSpark and N-DYKDDDDK (Flag) tag expression vector. (**A**) Western blot analysis of HEK293T cell lysates overexpressing wild-type ADNP-GFPSpark and mutated constructs using an anti-GFP antibody. (**B**) Western blot analysis of HEK293T cell lysates overexpressing wild-type ADNP-GFPSpark and mutated constructs using the N-terminal ADNP antibody (Aviva Systems). (**C**) Western blot analysis of HEK293T cell lysates overexpressing wild-type ADNP-DYKDDDDK (Flag) and mutated constructs using an anti-DYKDDDDK antibody. (**D**) Western blot analysis of HEK293T cell lysates overexpressing wild-type ADNP-DYKDDDDK and mutant constructs using the N-terminal ADNP antibody (Aviva Systems). The observed molecular weight of wild-type ADNP-GFPSpark is 175 kDa (including 25 kDa GFPSpark tag), respectively ADNP-DYKDDDDK 150 kDa, with each of their mutants showing a lower molecular weight as a consequence of the truncating mutations. Detection with antibodies for GFP, DYKDDDDK (Flag), and ADNP gave comparable results. GAPDH was used as a loading control in all experiments.

In addition to commercial ADNP antibodies, we next confirmed the molecular weight of 150 kDa by studying two expression clones of human recombinant ADNP with patient mutations in both an N-terminal GFPSpark and N-DYKDDDDK (Flag) tag expression vector fused to human ADNP transfected in HEK293T cells. In both cases, the GFPSpark and N-DYKDDDDK (Flag) tag ensure direct specific detection of ADNP. To validate whether mutant forms of ADNP are stably synthesized, site-directed mutagenesis of the human ADNP expression vectors was applied to evaluate effects of nonsense and frameshift stop mutations similar to Helsmoortel-Van der Aa syndrome patient mutations. Protein lysates of HEK293T cells transfected with either wild-type or mutated ADNP constructs were analyzed by immunoblotting for anti-GFP, anti-DYKDDDDK (Flag), and the N-terminal antibody of Aviva Systems, since Helsmoortel-Van der Aa mutations are characterized by a premature stop codon truncating the C-terminus of ADNP^14^. A GFP-positive signal was observed at 175 kDa in HEK293T cells transfected with the wild-type GFPSpark construct, while a DYKDDDDK-signal for ADNP was seen at 150 kDa (Fig. 7A/C). This dual approach confirms the previously observed 150 kDa signal for ADNP (see above), because GFP itself accounts for 25 kDa of the total molecular weight. The mutant forms of ADNP were observed at a lower molecular weight in the lysates of p.Tyr719*, p.Leu823Hisfs*6 and p.Asn832Lysfs*81 compared to a wild-type overexpression lysate, confirming the formation of a shorter mutant protein as an effect of the genetic mutation. The p.Arg173* nonsense mutation only appeared after immunoblotting of the GFP construct, but not in the DYKDDDDK-condition, perhaps due to stabilization of mutant ADNP by the larger GFP fusion protein, in line with our previous study which showed that N-terminal ADNP mutants are rapidly degraded by the proteasome^7^. The DYKDDDDK-antibody showed a lot more non-specific binding after immunoblotting and for this reason we repeated the experiment under the same conditions with the selected N-terminal ADNP antibody (Aviva Systems) to compare the results of the Western blot for anti-GFP and anti-DYKDDDDK. Since immunoblotting using an anti-fusion tag antibody allows unambiguous detection of ADNP, the observed signal is a reliable reference to validate the signals of the anti-ADNP antibody, which showed inconclusive results in in vitro sample materials (Fig. 7B/D). Both immunoblots show similar results, detecting wild-type ADNP at a molecular weight of 175 kDa (GFPSpark) and 150 kDa (DYKDDDDK Flag). In line, the mutant constructs are detected at a lower molecular weight of 45 kDa (p.Arg173*), 105 kDa (p.Tyr719*), 118 kDa (p.Leu823Hisfs*6), and 127 kDa (p.Asn832Lysfs*81). Respectively, the flag-tagged ADNP mutants are detected at 20 kDa (p.Arg173*), 80 kDa (p.Tyr719*), 93 kDa (p.Leu823Hisfs*6), and 103 kDa (p.Asn832Lysfs*81), accounting for a molecular weight difference of 25 kDa for GFP. In this section, we expressed N-GFPSpark and N-DYKDDDDK tagged human ADNP clones for immunoblot assessment, which further corroborated a molecular weight of 150 kDa for wild-type ADNP. Additionally, we were also able to detect mutant ADNP forms of both expression vector constructs with a lower molecular weight with antibodies recognizing the epitope tag as well as the N-terminal antibody (Aviva Systems), indicating mutant ADNP protein at cellular levels.

### Detection of heterozygous truncated ADNP mutants in a CRISPR/Cas9-engineerd HCT116 colon cancer cell line

**Figure 8:**
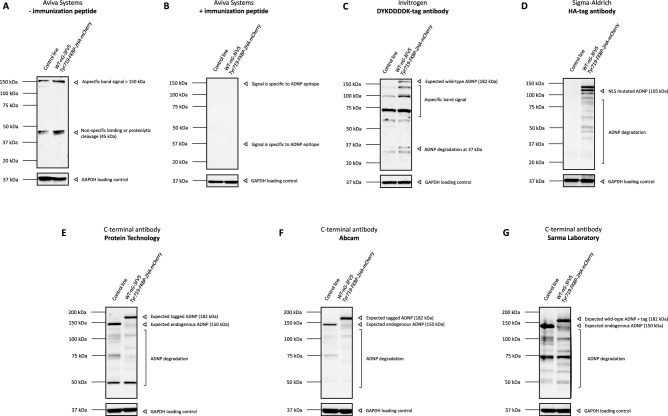
Western blotting of ADNP in a HCT116 colon cancer cell line, carrying the prevalent heterozygous p.Tyr719* mutation. HCT116 cells containing a wild-type and p.Tyr719* mutant allele were lysed in RIPA buffer and used as protein samples for the assessment with an N-terminal antibody, 3x-DYKDDDDK, HA-tag, and C-terminal ADNP antibodies with the optimized dilutions listed in Table 1. GAPDH was used as a loading control in all experiment. The predicted molecular weight of ADNP is 124 kDa. (**A**) The N-terminal antibody (Aviva Systems) recognizes ADNP in a range above its observed 150 kDa molecular weight an additional signal of 45 kDa, indicating proteolytic cleavage or non-specific binding. (**B**) Administration of the immunization peptide in a 5 × excess to antibody concentration reduced all signals, indicating that the N-terminal antibody does not bind ADNP specifically in HCT116 cells. (**C**) Detection of wild-type ADNP by means of the 3x-DYKDDDDK (Flag) epitope tag. Wild-type ADNP was detected in at 182 kDa in the 3xFlag-V5-loxP-neonGreen/3xHA-loxP-mCherry engineered line using a DYKDDDDK antibody, 32 kDa by tag insertion. (**D**) Detection of mutant ADNP by means of the HA-epitope tag. A truncated mutant p.Tyr719 ADNP protein was detected in at 105 kDa in the 3xFlag-V5-loxP-neonGreen/3xHA-loxP-mCherry engineered line using a HA-antibody, 25 kDa above its predicted molecular weight by tag insertion. Instability of the truncated protein was observed by a degrading smear. (**E–G**) Wild-type ADNP detection by means of three different C-terminal antibodies. Non-processed ADNP was detected with a strong signal at 150 kDa in the control line and at a molecular weight of 182 kDa in the genome-edited cell line. In both cases, a degrading smear was observed, indicating instability of the wild-type protein.

Previously, we have demonstrated stable translation of ADNP mutants in homozygous CRISPR/Cas9 endonuclease-mediated mESCs and an in an overexpression cellular model. However, these artificial systems do not approach the actual human disease condition e.g., *Adnp* homozygosity is embryonically lethal^1,31^ and Helsmoortel-Van der Aa syndrome patients are all unified by heterozygous *ADNP* mutations^13,14^, with endogenous ADNP protein expressed at nearly femtomolar concentrations^1,17^. For this reason, we further implemented a HCT116 colon cancer cell line, expressing an intact *ADNP* allele fused to 3xFlag-V5-loxP-neonGreen-STOP[-loxP], increasing the molecular weight by an additional 32 kDa together with a mutant allele coupled to 3xHA-loxP-mCherry-STOP[-loxP], attributing an additional 25 kDa. The mutant allele carried the most prevalent *ADNP* nonsense mutation, p.Tyr719*^14^. Expression of one intact wild-type allele and one *ADNP* mutant allele models the human condition rather accurately. Western blotting of total protein lysates of an unmodified HCT116 control and the *ADNP* heterozygous lines resulted in detection of a non-specific band above the molecular weight marker of 150 kDa. Additionally, expression of the *ADNP* mutant allele was not observed at a lower molecular weight of 105 kDa. Absence of the expected mutant went hand in hand with a non-specific band at 45 kDa (Fig. 8A). Administration of the blocking peptide caused complete loss of all observed signals after incubation with the N-terminal Aviva Systems antibody, proving this antibody is not suitable for reliable ADNP detection in HCT116 cells (Fig. 8B). To detect stable translation of wild-type and p.Tyr719* mutant ADNP, we implemented a western blot with incubation of a DYKDDDDK (Flag)-tag antibody, indicating the wild-type ADNP allele, and a HA-tag antibody for detection of the mutant protein. Using the DYKDDDDK (Flag)-tag antibody, we could show presence of wild-type ADNP approximately at 182 kDa amongst lower background signals (Fig. 8C). Detection of the p.Tyr719* mutation was mediated by incubation with a HA-tag antibody, showing presence of a truncated ADNP protein of 105 kDa with a lower degrading smear, indicating instability of the mutant protein (Fig. 8D). Since C-terminal antibodies do not detect the epitope for p.Tyr719* mutant recognition, we mainly detect endogenous and CRISPR-engineered wild-type ADNP in HCT116 mutant and control cell lines. Here, we could detect endogenous ADNP at a molecular weight of 150 kDa in the control cell line. Moreover, the modified *Adnp* wild-type allele shows expression of an intact ADNP protein, 32 kDa above its observed molecular weight of 150 kDa as detected by each of the three different C-terminal antibodies. Degrading signals were observed at lower molecular weights (Fig. 8E-G). Altogether, we show evidence for detection of a truncated mutant ADNP protein at lower molecular weight in an *Adnp* heterozygous colon cancer cell line. In line with previous observations, we detected endogenous ADNP levels at a molecular weight of 150 kDa, confirming an immunoblot signal above its theoretical mass.

### Lack of detectable mutant ADNP protein after immunoblot analysis in patient-derived sample materials

**Figure 9:**
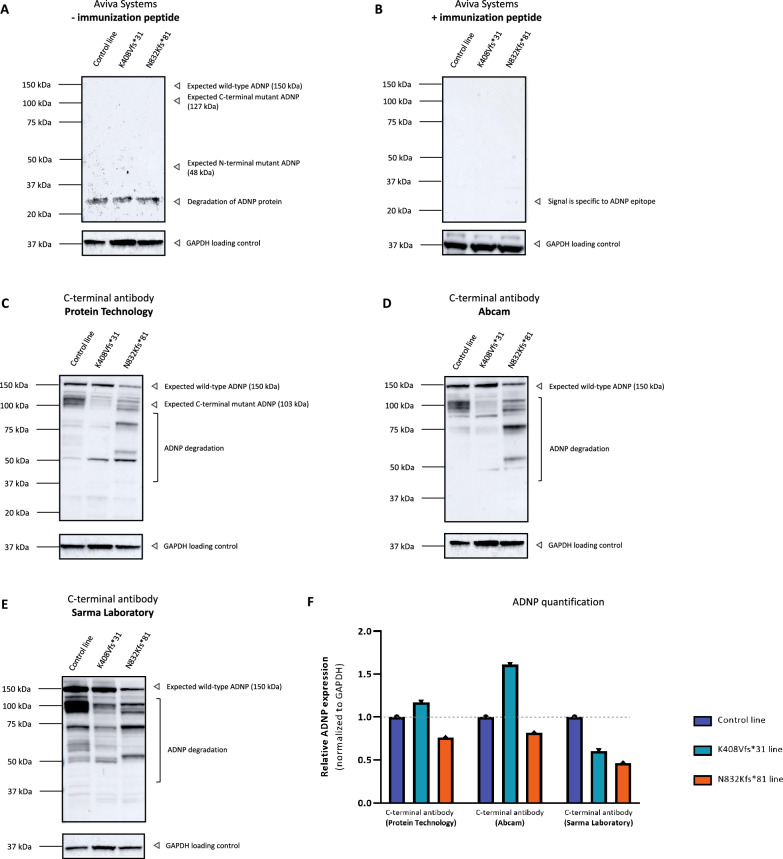
Western blotting of ADNP in human induced pluripotent stem cells (hiPSCs), carrying distinct heterozygous *ADNP* mutations mediated by CRIPSR/Cas9. (**A**, **B**) hiPSCs were lysed in RIPA buffer and analyzed by western blotting with the N-terminal antibody (Aviva Systems) with application of our blocking peptide competition assay. Here, no reliable ADNP signal was detected. The molecular weight of the *ADNP* mutant lines is expected to decrease to 127 kDa for the Asn832Lysfs*81, respectively to 48 kDa for the lys408Valfs*31 line. However, no signal is observed at the predicted weight for the mutations. (**C**–**E**) The C-terminal antibodies of Protein Technology, Abcam, and the Sarma Laboratory were able to visualize wild-type ADNP at 150 kDa. Possessing the desired epitope for mutant ADNP detection, the C-terminal antibody of Protein technology was not able to capture the predicted truncated protein. GAPDH was used as a loading control. (**F**) The ADNP signal was quantified determining the ratio of the wild-type protein in mutant to control cell lines. Here, the relative ADNP expression decreased in the Asn832Lysfs*81 cell line compared to the control, whereas mutant-to-wild-type expression ratio showed a higher signal with the antibodies of Protein Technology and Abcam in the lys408Valfs*31 cell line.

Our artificial expression systems allowed assessment of the validity of the blocking peptide strategy, and it also provided evidence on the effect of *ADNP* mutations at the protein level. To determine whether truncated ADNP is retained in patients carrying the heterozygous N-terminal lys408Valfs*31 and C-terminal Asn832Lysfs*81 mutations, we examined ADNP protein levels in their corresponding mutant hiPSCs compared to an unrelated control. We used ADNP antibodies that specifically recognize a region within the N-terminus (Aviva Systems) and C-terminus (Protein Technology, Abcam, and Sarma Laboratory) of ADNP. Western blot analysis showed that a truncated fragment with a theoretical molecular weight of 48 kDa for the lys408Valfs*31 mutation, respectively 127 kDa for the Asn832Lysfs*81 mutation together with a wild-type ADNP signal could not be detected after N-terminal antibody incubation. The N-terminal ADNP antibody only recognized an epitope at the molecular weight of 25 kDa, suggestive for degradation (Fig. 9A). The signal of 25 kDa disappeared after administration of our blocking peptide competition assay, suggesting recognition of an ADNP epitope in the degraded signal (Fig. 9B). All C-terminal antibodies recognize wild-type ADNP at a molecular weight of 150 kDa with a degrading smear, confirming previous observations in our artificial overexpression model as well as in cell lines and tissue samples (Fig. 9C–E). Interestingly, the C-terminal antibody of Protein technology was raised against aa757-1102 and can therefore detect a possible Asn832Lysfs*81 mutant protein with an expected molecular weight of 127 kDa. Again, no mutant ADNP protein could be detected (Fig. 9C). Signal quantification of ADNP in mutant to control cell lines normalized by GAPDH resulted in a decreased relative expression for the C-terminal Asn832Lysfs*81 mutation for all three antibodies, but not for the lys408Valfs*31 mutation where the mutant-to-wild-type expression ratio showed a higher signal with the antibodies of Protein Technology and Abcam (Fig. 9F).

**Figure 10:**
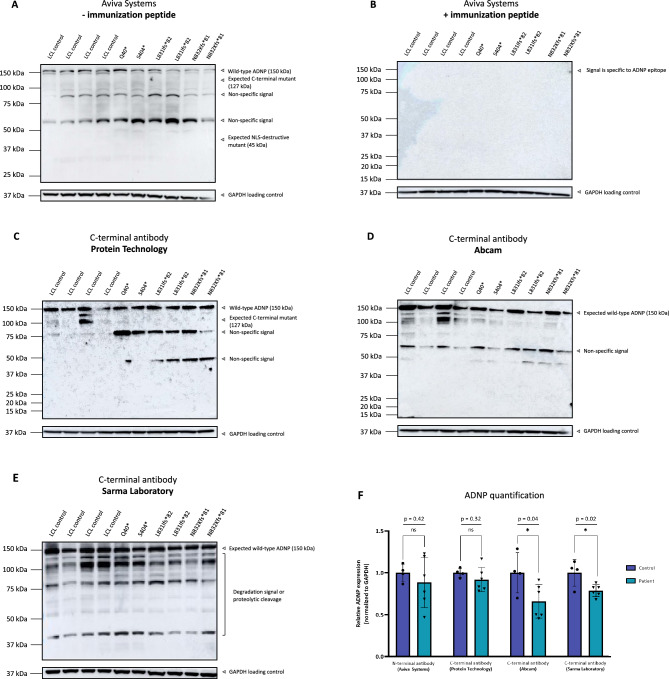
Absence of a mutant ADNP protein after immunoblotting of different lymphoblastoid cell lines from Helsmoortel-Van der Aa syndrome patients. (**A**) LCLs of four control subjects and six patients were lysed in RIPA buffer and analyzed by western blotting with the N-terminal antibody (Aviva Systems). The expected wild-type ADNP signal presented at 150 kDa together with two non-specific bands at 50 kDa and 75 kDa with no difference in expression (p = 0.42; ns) of the wild-type protein. However, the ADNP mutants at a lower molecular weight of 127 kDa for the Asn832Lysfs*81 and Leu831Ilefs*82 mutations, respectively to 45 kDa for the Ser404* mutation, and to 10 kDa for the cell line carrying the Gln40* mutation could not be visualized. (**B**) Administration of the immunization peptide in a 5 × excess to antibody concentration reduced all signals, indicating that the N-terminal antibody recognized ADNP specifically in LCLs alongside non-specific band signals. (**C-E**) C-terminal antibodies detected wild-type ADNP at a molecular weight of 150 kDa. No mutant ADNP was observed with the antibody of Protein Technology which is capable to recognize a part of the truncated Asn832Lysfs*81 and Leu831Ilefs*82 mutations. (**F**) All C-terminal antibodies visualized wild-type ADNP at 150 kDa, with only the Abcam (p = 0.04; *) and Sarma Laboratory (p = 0.02; *) antibodies showing the expected reduction of ADNP in LCLs of Helsmoortel-Van der Aa syndrome patients. GAPDH was used as a loading control.

In order to further investigate the presence of endogenous mutant ADNP, we performed western blotting experiments with the same antibody panel in LCLs of four healthy subjects and six ADNP patients carrying distinct mutations spread all over the protein, e.g., Q40*, Ser404*, Leu831Ilefs*82, and Asn832Lysfs*81. First, all three C-terminal antibodies were able to detect wild-type ADNP at a molecular weight of 150 kDa. The antibody of Protein Technology, raised against aa757-1050 and thus in part able to recognize the mutant proteins formed of the p.Leu831Ilefs*82 and p.Asn832Lysfs*81 *ADNP* mutations, did not show a truncated ADNP protein with a predicted molecular weight of 127 kDa, and solely detected expression of the wild-type protein with no difference (ns, *p* = 0.32; student T-test) in ADNP levels in LCLs of patients compared to healthy subjects. Moreover, two strong non-specific bands were observed at 75 kDa and 50 kDa, as previously shown in mESCs (Fig. 10C**/F**). The other C-terminal antibodies do not contain the necessary epitope for mutant ADNP recognition and showed a significant decrease in the expression of ADNP (*p = 0.04; student T-test) with the C-terminal antibody of Abcam and (*p = 0.02; student T-test) with the C-terminal antibody of the Sarma Laboratory in LCLs of patients compared to healthy controls (Fig. 10C-F). On the other hand, the N-terminal antibody recognized a wild-type ADNP signal at 150 kDa alongside other protein bands at lower molecular weights, showing no difference in the expression (ns, *p* = 0.42; student T-test) in the patient LCLs compared to the control lines (Fig. 10A). The wild-type ADNP signal at 150 kDa together with the non-specific lower band signals disappeared after administration of the blocking peptide (Fig. 10B). Surprisingly, no mutant proteins could be detected in the *ADNP* heterozygous LCLs from different patients after N-terminal antibody incubation. Moreover, non-specific band signals at 50 kDa and 75 kDa were observed in all LCLs with a stronger intensity in patient lines. In contrast to the specific ADNP signal in murine, rat and human brain sections, we report that the N-terminal antibody recognizes a specific ADNP signal alongside non-specific band signals in lymphoblastoid cell lines, which can be attributed to the higher signal-to-noise ratio in brain versus lymphoblastoid model systems. Taken together, western assessment of different patient-derived sample materials showed no detectable mutant ADNP protein levels irrespective of the selected antibody, suggestive for degradation and/or absence of a mutant ADNP protein in the Helsmoortel-Van der Aa syndrome.

### Enrichment of mutant ADNP proteins in HEK293T overexpression lysates and patient-derived sample materials.

**Figure 11:**
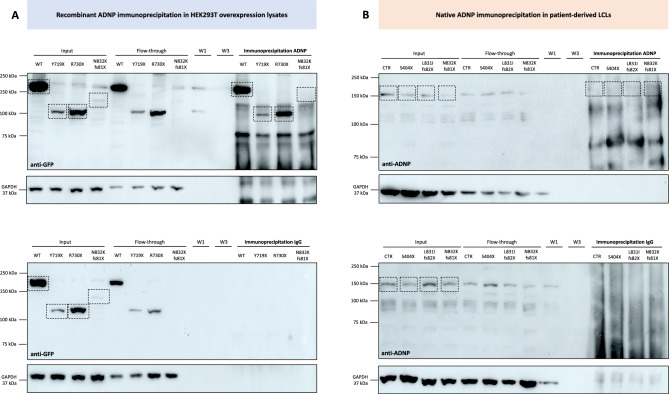
Wild-type and mutant ADNP enrichment through immunoprecipitation. The N-terminal sc-F5 ADNP IP-competent antibody was crosslinked to agarose beads and sequentially eluted in fractions (input; flow-through; three consecutive washes, W1-W3; and the immunoprecipitated fracted. IgG non-reactive beads were used as a negative control. In each lane, 20 μg of protein was separated by SDS-PAGE electrophoresis. GAPDH has been used as loading control for all western blots, and critical assessment of the accuracy of the IP method. (**A**) Immunoprecipitation assay of recombinant wild-type (WT) ADNP and truncating mutants (p.Tyr719*; p.Arg730*; p.Asn832Lysfs*81) in HEK293T overexpression lysates. (**B**) Immunoprecipitation assay of native wild-type (WT) ADNP and truncating mutants in protein extracts of LCLs derived from a control subject (CTR) and patients with the p.Ser404*, p.Leu831Ilefs*82, or p.Asn832Lysfs*81 *ADNP* mutation.

*ADNP* mutations in Helsmoortel-Van der Aa syndrome are of heterozygous nature, and therefore presence of both wild-type and truncated ADNP could promote competition, thereby impacting protein detection levels^7^. To explore presence of a truncating ADNP protein or its wild-type counterpart, we enriched HEK293T overexpression lysates and extracts prepared from patient-derived LCLs using an immunoprecipitation (IP)-competent N-terminal sc-F5 ADNP antibody, which was previously shown to capture wild-type and mutant ADNP together with other SWI/SNF interactors in HEK293T cells^1^. During this process, we performed stringent washing steps using high detergent buffers to prevent false-positive protein binding. In addition, we also controlled each western blot with GAPDH, whose intensity was absent after immunoprecipitation of the bait protein. Immunoprecipitation with the IP-competent N-terminal ADNP antibody in HEK293T overexpression lysates resulted in specific detection of recombinant ADNP-GFP (175 kDa) together with the GFP-mutants p.Tyr719* (105 kDa) and p.Arg730* (106 kDa), whereas no immunoreactivity was observed for the p.Asn832Lysfs*81 (127 kDa) mutant after GFP antibody incubation. IgG non-reactive beads, used as a negative control, showed no immunoreactivity in the eluted fractions (Fig. 11A). In this respect, we employed the exact same immunoprecipitation strategy in protein extracts of patient-derived LCLs. Here, immunoprecipitation with the same IP-competent ADNP antibody resulted in detection of native ADNP (150 kDa) in control and patient cell lines in presence of the polyclonal N-terminal ADNP antibody (Aviva systems), whereas no mutant protein could be enriched for the patients with the p.Ser404* (45 kDa), Leu831Lysfs*82 (103 kDa), and p.Asn832Lysfs*81 (103 kDa) mutation. Again, IgG non-reactive beads were used as a negative control and indicated no immunoreactivity in the eluted fractions (Fig. 11B). Taken together, our protein enrichment strategy using immunoprecipitation was successful in capturing wild-type ADNP and various truncating variants in HEK293T overexpression systems but failed to enrich a native mutant protein in patients with Helsmoortel-Van der Aa syndrome.

## Discussion

In this study, we compared and optimized several strategies for the detection of ADNP in different specimens with multiple ADNP antibodies. The rationale for our search was the occasional ambiguous ADNP signals detected with various antibodies reported in literature. For this reason, we validated a new N-terminal ADNP antibody (Aviva Systems) by means of a blocking peptide competition assay in cell lines. By this approach, we could identify a clear ADNP specific signal of 150 kDa in HEK293T, HeLa, and SH-SY5Y cells. In control LCL, this signal was much harder to detect. Additionally, we challenged the N-terminal ADNP antibody to distinct tissue samples, proving unambiguous ADNP detection with a molecular weight of 145–150 kDa. Inclusion of several commercialized C-terminal antibodies confirmed the specific 150 kDa signal, representative for ADNP, in different cell lines and tissue specimens, although immunoblot smears of smaller proteins bands using the C-terminal antibodies suggest some instability or degradation of the protein. The 150 kDa signal, was not detected after immunoblotting of protein lysates from a homozygous *Adnp* knock-out cell line, whereas the additional smaller bands remained. However, we were not able to detect a native mutant ADNP protein after immunoblotting of hiPSCs and LCLs derived from different patients, nor by protein enrichment using immunoprecipitation. In contrast, we were able to detect different ADNP mutants using both N-GFPSpark and N-DYKDDDDK (Flag) human recombinant expression vectors, as well as pull-down these variants by immunoprecipitation, again highlighting the discrepancy between recombinant and endogenous model systems. Additionally, in CRISPR/Cas9-engineerd homozygous mESCs and heterozygous HCT116 cells, ADNP mutants could also be detected by means of their epitope tag. These data suggest a potential stabilizing effect of the recombinant tags fused to ADNP.

Previous studies concerning ADNP have been focusing on detection of wild-type protein^5,6, 19, 20, 27^. With a theoretical molecular weight of 124 kDa, we also studied wild-type ADNP in different cell lines and tissue samples of mouse, rat, and human origin, revealing an observed molecular weight around 150 kDa. This observation indicates that ADNP undergoes post-translational modifications such as phosphorylation, SUMOylation, methylation, acetylation, and ubiquitin conjugation after synthesis (Uniprot; Q9H2P0). Besides a higher molecular weight than predicted, we also demonstrated protein instability as witnessed by a degrading smear as well as presumed proteolytic cleavage bands in tissue samples. The latter observation is in line with the fact that ADNP contains potential dibasic processing sites such as KKRK (aa 447–450) and KRKK (aa 526–529)^6^. In 2017, foot-and-mouth disease virus leader protease (L^pro^), a protein involved in innate immunity, was found to bind ADNP in vitro to complex with ADNP and chromatin remodeler BRG1, modulating gene expression. In this study, ADNP was processed or degraded during infection: western blotting experiments showed ADNP at a molecular weight of 150 kDa together with discrete band signals at lower molecular weights. To prove this concept, bacterial cell extracts containing wild-type and enzymatic inactive L^pro^ were administered to HEK293T cells overexpressing human recombinant ADNP. Here, ADNP expression decreased with active L^pro^ and revealed a cleavage product at 120 kDa. No processing was detected with the enzymatic inactive control. These results indicate processing of ADNP by proteases^33^. Recently, a computational analysis identified ADNP cleavage sites mediated by caspases and proprotein convertases^34^. Amino acid residues 734–738 of ADNP, residing at the C-terminal part of the protein, contain the motif DDSDS, which is potentially recognized by caspase-3 and caspase-7. These caspases, especially caspase 3, are important players in the canonical apoptotic pathway^35^. When cleaved at the predicted site, ADNP has a theoretical length of 737 aa contributing to a molecular weight of 82 kDa, a band signal we also observe in human samples. Interestingly, a prevalent *ADNP* nonsense mutation p.Arg730* resides near this predicted caspase cleavage site. Additionally, the somatic frameshift mutation p.Arg730Thrfs*4 truncates to protein of the same length. Besides involvement of caspase activity, proprotein convertases also play a role in proteolytic processing of neuropeptides such as ADNP. For example, the 367–371 aa motif KQLLP is a substrate for members of the proprotein convertase family. After cleavage, a fragment of 367 aa of ADNP is formed, contributing to a molecular weight of 41 kDa which corresponds with the fragment formed after the frameshift mutation p.Ile359Thrfs*8 found in a post-mortem Alzheimer brain^34^. We also observed a 40 kDa signal of ADNP in human post-mortem brain samples, indicative of degradation of the protein.

In our search to verify the molecular weight of the ADNP protein, we designed a different strategy to assess the stability of ADNP expression by means of a CRISPR/Cas9 *Adnp* knockout cell line and two expression vectors, containing either an N-terminal GFPSpark or an N-DYKDDDDK (Flag)-tag. Homozygosity of *Adnp* in mESCs resulted in disappearance of the 150 kDa signal, indicating this signal is unique to wild-type ADNP. Next, we used the signal of the epitope-tag and detected wild-type ADNP at a molecular weight of 150 kDa (Flag) and 175 kDa (GFPSpark) in overexpression lysates. These findings reveal again a molecular weight of 150 kDa for ADNP, confirming that the observed signal in mouse, rat and human samples is a reliable and unique specific signal. Additionally, western blotting with anti-GFP and anti-DYKDDDDK (Flag) antibodies revealed presence of mutant ADNP forms after site-directed mutagenesis in our expression vector system. Whereas a previous study has already indicated proteasomal decay of unstable shortened ADNP proteins upon introduction of patient-specific mutations in our expression vector construct residing towards the N-terminal portion of the protein, it might be possible that conservation of the NAP domain, which was shown to account for the neuroprotective function of the protein, is essential for ADNP protection against proteolytic degradation^7^. Remarkably, comparison of the p.Arg173* mutation in our overexpression system resulted in stable translation in the GFPSpark condition but not in the N-DYKDDDDK (Flag) clone, indicating that a larger epitope tag helps stabilization of ADNP mutants. In fact, detection of ADNP mutants in homozygous mESCs and heterozygous HCT116 cells was facilitated by recognition with an antibody against the epitope tag. However, western blotting of these mutants, endogenous to patient-derived sample materials, showed complete absence of a signal, thereby suggesting that addition of an external tag helps with protein stabilization. Recently, a study was published where *Adnp* was CRISPR/Cas9-edited in a N1E-115 mouse neuroblastoma cell line, expressing either wild-type *Adnp* or truncated ADNP both fused to a GFP tag. Western blotting with a GFP antibody clearly shows the wild-type protein and truncated mutants at lower molecular weights. However, detection with an N-terminal antibody showed multiple immunoreactive bands starting from 175 kDa and rather indicated a degrading ADNP smear than proper detection of a stable mutant protein^25^. In line with our results in homozygous and heterozygous genome-edited ADNP-expressing cell lines, we were able to detect truncated ADNP mutants at a lower molecular weight after incubation with an epitope-tag antibody, whereas results with an N-terminal antibody were inconclusive. Previously, we have demonstrated the presence of intact mutant mRNA in Helsmoortel-Van der Aa syndrome patients, indicative for escape from nonsense-mediated decay (NMD)^14^. However, patient-derived materials show no evidence of the mutant protein as observed in our expression genome-edited cell lines and vector systems, suggesting that NMD cannot be totally excluded as there are many post-transcriptional processes involved in mRNA processing to the resulting protein product. Indeed, there are genes containing mutations in the last exon such as hexoseaminidase A (HEXA), myelin protein zero (MPZ) and the T-cell receptor (TCR) which are exceptions on the rule of NMD, normally only occurring with mutations near exon-exon junctions after recognition of a premature stop codon by the exon-junction complex^36^. However further research towards post-transcriptional regulation of ADNP expression in patient-derived sample material should be conducted to fully exclude this hypothesis.

Next to detection of wild-type ADNP, either cleaved or degraded ADNP was observed at lower molecular weights. With respect to proteolytic cleavage, we need to further address the signal observed at 85 kDa with the N-terminal antibody (Aviva Systems) in human brain lysates that could potentially be cleaved or indicate an unknown isoform. Next, western blotting of mouse and rat brain lysates with C-terminal ADNP antibodies also revealed both 50 kDa and 65 kDa signals which also was detected in protein lysates of human brains. Here, once again, our Helsmoortel-Van der Aa syndrome patient carrying the heterozygous de novo *ADNP* mutation c.1676dupA/p.His559Glnfs*3 showed no truncated protein after western blotting. At first sight, these observations seem to be in contrast with our results obtained with the *ADNP* expression vector. However, since ADNP expression vectors lack some 3’UTR regulatory sequences, we cannot exclude that artificial overexpression systems do not completely mimic the endogenous regulation of ADNP protein translation or stability^37^. As such, lack of some negative regulatory 3’UTR sequences in the expression vectors may contribute to the possible detection of ADNP protein in HEK293T cells. Nevertheless, our results also revealed critical involvement of proteolytic cleavage of ADNP by proteases or alternative splicing in vivo.

Surprisingly, we were not able to detect mutant ADNP after western blotting of either post-mortem cerebellar brain tissue, hiPSCs, or LCLs carrying an *ADNP* mutation. We propose three possible explanations: (1) the mutation might affect folding and ubiquitination of the protein, resulting in faster processing of the mutant protein compared to wild-type ADNP^38^, (2) early evidence shows a reciprocal role of ADNP in regulation of transcription and translation and for this reason we could hypothesize that mutations affect the translation machinery, thereby impacting the final levels of mutant ADNP^39^. Therefore, a mutant protein might still be present, but below the detection limit for western blotting, and (3) the signal-to-noise ratio in different protein extract specimens, since ADNP is highly expressed in neuronal tissues^1^ but contains for example low expression in lymphoblastoid cell lines, causing the signal-to-noise ratio to be higher in brain tissue, resulting in detection of more non-specific epitopes in lymphoblastoid protein extracts due to higher background signal intensity.

Alternatively, it is possible that the mutant protein is simply not present. This would predict haploinsufficiency as a disease mechanism. However, such a mechanism is barely compatible with the genotype–phenotype correlation that has now been firmly established^40^. Patients with a mutation in the central region of the gene are more severely affected than patients with a mutation near the C- or N-terminal regions of ADNP^40^. These two disease-severity classes overlap with different and partially opposing genome-wide episignatures depending on the mutation location^41,42^, and were also reflected in different *Adnp* mouse models, where phenotypic and molecular differences were observed between haploinsufficient and Tyr718* heterozygous mice^43^. The absence of detectable mutant protein contrasts with the suggested higher expression on mutant mRNA as compared to the wild-type alle^2^.

In summary, we demonstrated evidence for the presence of mutant forms of ADNP in our HEK293T overexpression system for a couple of useful antibodies with evidence for reliable detection. We were not able to detect native ADNP mutants in patient-derived sample materials, but we can also not completely exclude presence at concentrations below the antibody detection threshold and recommend targeted proteomic-based methods to completely exclude this hypothesis. Our western blot assessment has revealed that a dual antibody approach is required for reliable ADNP detection and that the actual molecular weight of ADNP is expected at 150 kDa rather than the theoretical molecular weight of 124 kDa. Together, this study shapes awareness for critical western blot assessment of ADNP specific protein variants with the eye on future functional studies by combined antibody-based detection methods in order to unravel the mutational mechanism underlying the Helsmoortel-Van der Aa syndrome.

### Supplementary Information

Supplementary Figures.

## Data Availability

The data presented in this study is available in the article or contained in the supplementary information.
